# Country Reports on Practical Aspects of Conducting Large-Scale Community Studies of the Tolerability of Mass Drug Administration with Ivermectin/Diethylcarbamazine/Albendazole for Lymphatic Filariasis

**DOI:** 10.4269/ajtmh.21-0898

**Published:** 2022-03-15

**Authors:** Purushothaman Jambulingam, Swaminathan Subramanian, Kaliannagounder Krishnamoorthy, Taniawati Supali, Peter Fischer, Christine Dubray, Carl Fayette, Jean Frantz Lemoine, Moses Laman, Christopher King, Josaia Samuela, Myra Hardy, Gary J. Weil

**Affiliations:** ^1^ ICMR-Vector Control Research Centre, Puducherry, India;; ^2^ Universitas Indonesia, Jakarta, Indonesia;; ^3^ Washington University in St. Louis, St. Louis, Missouri;; ^4^ Centers for Disease Control and Prevention, Atlanta, Georgia;; ^5^ IMA World Health, Pétion-Ville, Haiti;; ^6^ Ministère de la Santé Publique et de la Population, Port-au-Prince, Haiti;; ^7^ Papua New Guinea Institute of Medical Research, Madang, Papua New Guinea;; ^8^ Case Western Reserve University, Cleveland, Ohio;; ^9^ Fiji Ministry of Health and Medical Services, Suva, Fiji;; ^10^ Murdoch Children’s Research Institute, Melbourne, Australia

## Abstract

This article is a compilation of summaries prepared by lead investigators for large-scale safety and efficacy studies on mass drug administration of IDA (ivermectin, diethylcarbamazine, and albendazole) for lymphatic filariasis. The summaries highlight the experiences of study teams that assessed the safety and efficacy of IDA in five countries: India, Indonesia, Haiti, Papua New Guinea, and Fiji. They also highlight significant challenges encountered during these community studies and responses to those challenges that contributed to success.

## INTRODUCTION

**Figure 1.**
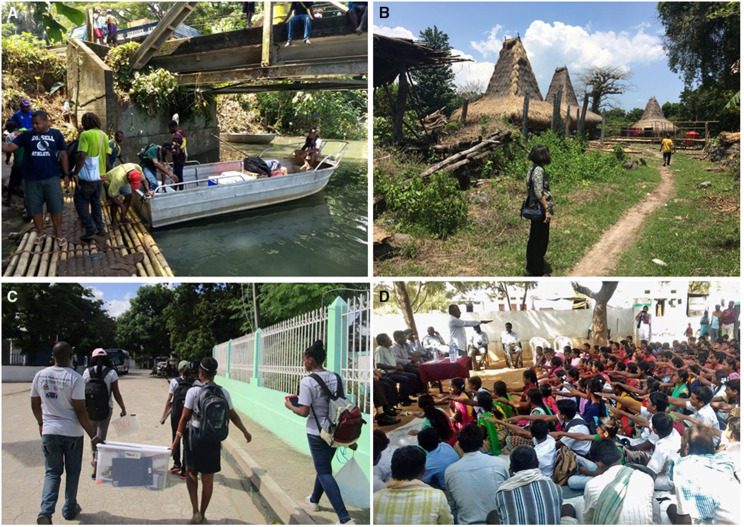
Access to study areas. (**A**) Boats and footbridges were required to transport people and supplies in Bogia (Papua New Guinea). (**B**) Some of the study villages in Sumba Barat Daya (Indonesia) were only accessible by foot paths. (**C**) The semiurban study area in Quartier Morin (Haiti) was semiurban and easily accessible. However, it was sometimes difficult for the study team to correctly identify participants’ houses and neighborhood boundaries. (**D**) The Yagdir study area in India is densely populated. This photo shows a community leader asking school-aged children to affirm that they would comply with mass drug administration.

**Figure 2.**
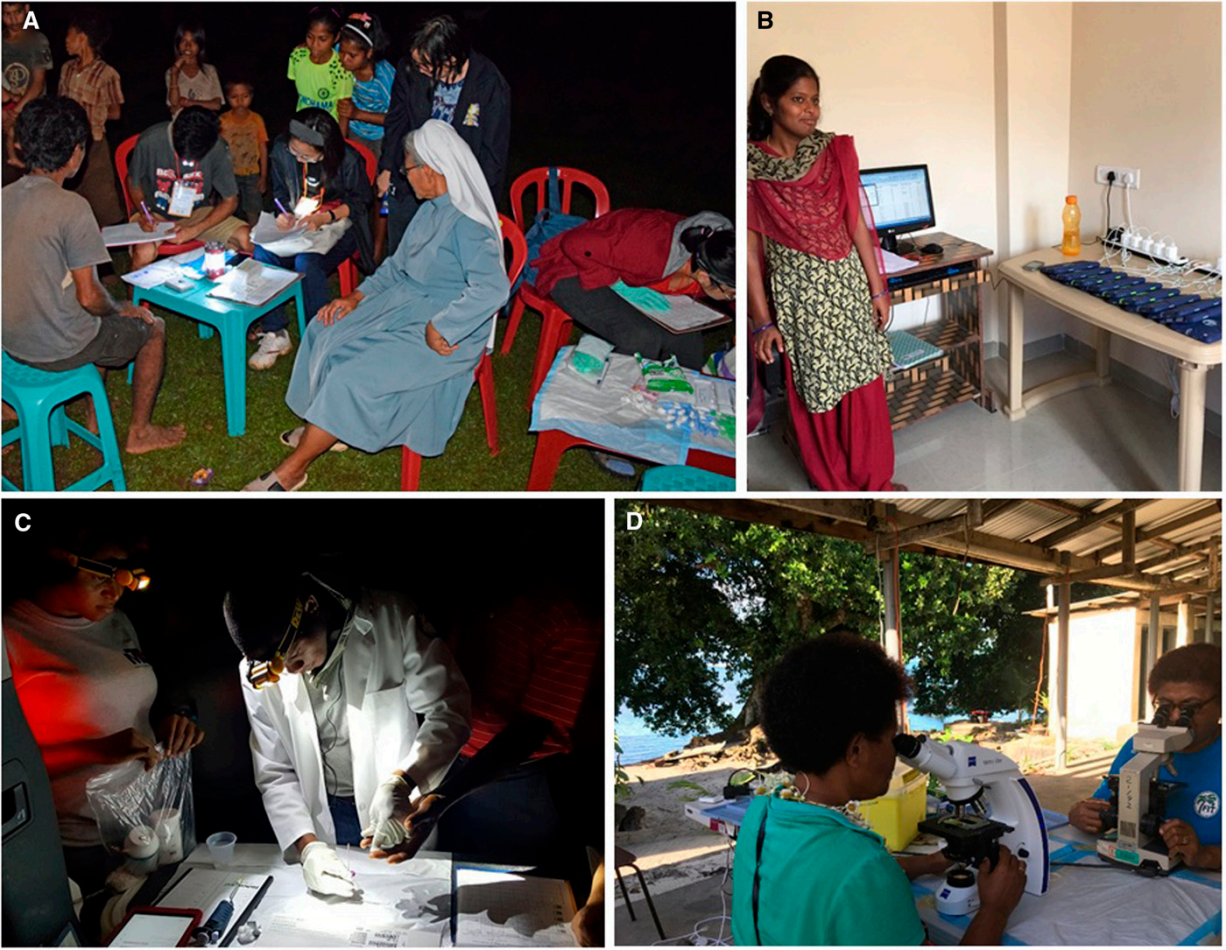
Field surveys and laboratory support. (**A**) A locally resident nun witnessed registration of study participants at night in Indonesia. Inclusion of trusted local residents in the study team helped to improve community participation. (**B**) A data manager in India charges tablet computers needed for data collection in study villages. (**C**) Field teams used headlamps to collect night blood samples in Haiti. (**D**) Microscopists in Fiji enjoy a gentle ocean breeze as they search for microfilariae in stained thick blood smears.

Clinical trials conducted by the Death to Onchocerciasis and Lymphatic Filariasis (DOLF) Project ^1^ showed that a new triple drug combination therapy (ivermectin plus diethylcarbamazine and albendazole [IDA]) was superior to the established treatment regimen of diethylcarbamazine and albendazole (DA) for the treatment of lymphatic filariasis (LF). ^2^^,^ ^3^ The new regimen had the potential to be a game changer for the Global Program to Eliminate LF, which distributes medicines to more than 400 million people in LF-endemic countries each year. ^4^ The clinical trial results were a catalyst to accelerate IDA’s further clinical development. To support a recommendation from the World Health Organization (WHO), additional evidence was required on the safety, efficacy, population acceptability, and operational feasibility of IDA in different country contexts. Planning for the further clinical development of IDA is described in another article in this Supplement. This article highlights practical issues related to the conduct of large-scale studies in five countries (India, Haiti, Indonesia, Papua New Guinea, and Fiji) that compared the tolerability and efficacy of IDA versus DA. ^5^ The lead investigators for each study site prepared summaries that focus on practical issues and the many activities that were required to complete the study in a timely manner. The summaries also highlight significant programmatic challenges and responses to those challenges that contributed to success. Figures 1 and 2 contain photos from each of the study sites that illustrate points made in the text. The authors hope that these accounts and lessons learned will help others who will conduct similar community studies of new interventions to control or eliminate neglected tropical diseases.

## INDIA

### Background and preparation for field studies.

The Indian Council of Medical Research (ICMR, under the Indian Ministry of Health and Family Welfare) was keen to perform an IDA community trial for LF in India, based on the results of clinical trials in other countries. That is because India accounted for approximately one third of the global LF burden per WHO at that time. ^6^ In 2016, the Director General of ICMR encouraged and supported the early launch of a large safety and efficacy study to determine whether this treatment might be appropriate for wide use in India. However, various stakeholders and subject experts were initially skeptical about this project because of the large number of tablets of medicine individuals would be required to take for IDA, and they predicted that community acceptance and compliance would be low.

The ICMR selected the Vector Control Research Center (VCRC, based in Puducherry, Tamil Nadu) to conduct the trial based on its long experience in filariasis research at the community level and its expertise in different disciplines including social science. ICMR-VCRC is a WHO Collaborating Center for Research and Training in LF and for Integrated Vector Management. The National Vector Borne Diseases Control Program (NVBDCP) was brought on board to work on the study in early 2016 based in part on high-level advocacy from ICMR, WHO, and LF experts. The study proposal was reviewed by expert committees at VCRC, ICMR, NVBDCP, and the National Task Force on LF elimination in India (chaired by a former director general of ICMR). After considering various options, the NVBDCP recommended Yadgir district in Karnataka state as an appropriate site for the study.

The DOLF Project provided technical support throughout the study; financial support came from the Bill & Melinda Gates Foundation (Seattle, WA) and the Task Force for Global Health (Decatur, GA). A technical advisory committee (Ministry of Health and Family Welfare, Government of India) conducted final reviews and approved the study. After receiving approval at the national level, the Ministry of Health and Family Welfare in Karnataka state also reviewed and approved the proposal. Project leaders discussed the study’s goals and plans in detail with district administration and health officials of Yadgir district before implementation. Dr. V. Kumaraswamy, a technical expert on LF, took the lead on arranging many of the preliminary administrative reviews at different levels. His sudden demise in early 2016 was a terrible blow (professionally and personally) that set back the research for some time.

India requires strict adherence to regulatory processes for conducting drug-based intervention studies. The regulatory process required approval from the Ministry of Health and Family Welfare, approval for the use of ivermectin from the Drug Controller General of India (received in February 2016), approval for importation of donated drugs (received in May 2016), and exemption from customs duty for donated drugs (received in May 2016). Following ICMR guidelines, the Institutional Human Ethics Committee of ICMR-VCRC approved the study protocol after ensuring compliance with required provisions such as insurance coverage for the participants (received in June 2016). The proposal also required approval from the Health Ministry’s Screening Committee for obtaining foreign financial support and collaboration for the study. The final step was registration of the study with the Clinical Trial Registry of India (completed in October 2016). Active support and intervention by high-level officials enabled the study trial to pass through many regulatory hoops in a relatively short time (approximately 10 months).

Recruitment and appointment of project staff in compliance with the rules and regulations from the government of India and ICMR was quite challenging. Responses to published advertisements were initially disappointing. However, with support from the local district health office and nursing colleges, we were eventually able to recruit the required number of technical and support staff. Recruiting physicians for these short-term positions was much more difficult. Local physicians were generally unable to suspend their practices to participate in a short-term research project. We had initially proposed 3 months to complete our target recruitment enrollment of study participants (∼10,000), but this was not possible due to the smaller number of treatment teams (8 versus 15) because of a shortage in medical personnel. Consequently, it took 5 months to complete enrollment, treatment, and safety assessments.

The ICMR-VCRC placed a senior-level scientist/physician to be stationed in Yadgir district throughout the study to coordinate field activities and also recruited several recent medical graduates who were awaiting postgraduate appointments.

The research team was also strengthened by the addition of a retired senior physician with extensive clinical trial experience. The addition of these staff members complemented the capabilities of the ICMR-VCRC technical team and were important to ensuring the study’s success.

### Implementation of field studies.

Training was conducted in the town of Yadgir with field visits, and the study was initiated in early October 2016. Support from the state health department facilitated cooperation across different sectors. The study was closely monitored by the ICMR-VCRC Institutional Human Ethics Committee, Data Safety Monitoring Boards (national and international), and a steering committee that was constituted to advise the study team. A dedicated and active program officer at ICMR headquarters in New Delhi played a major role in organizing the review meetings, obtaining approvals at ICMR, and providing logistical support to the field team. Training and guidance provided by DOLF project leaders and staff (in Yadgir and remotely), along with the support from Gates Foundation, were extremely helpful for initiation and management of the study. A contract research organization (CRO) based in Bangaluru (the capital of Karnataka state) was hired to help the study team with data management and compliance with good clinical practice (GCP) guidelines. We were also fortunate to have the strong support of the Vector Borne Disease Control Officer for Yadgir district who participated in all village meetings and helped mobilize the support of primary health center staff. Including physicians on each drug distribution team was also helpful, because they provided advice and referrals to participants regarding other medical conditions independent of LF or this study.

We selected Yadgir district for this study in part because of its known high prevalence of LF infection. Infections had persisted even after 15 rounds of mass drug administration (MDA) with either DEC alone or DEC with albendazole. Such persistence is a strong indicator of program fatigue with poor adherence to MDA. We anticipated these factors to be the major challenges in our study and therefore implemented social mobilization strategies to help overcome them. Before the treatment teams began making their daily visits, a social mobilization team from VCRC visited the area, enumerated the houses, and prepared the families by providing information about the MDA program. We also enlisted help from village advisory boards specifically created to promote adherence. Board members from the first study village visited other villages to share their experiences and motivate them to welcome study teams. Community heads and other highly respected village residents often accompanied our treatment teams.

Even with these social mobilization strategies in place, we sometimes encountered community resistance. For example, some community members insisted that all members of the research teams must be local residents. We responded by explaining that locally recruited staff would be part of the village research teams, but that we also required participation from outside experts and medical personnel who had specific skills and expertise required for community treatment trials. Support from district health personnel was especially useful for enrolling families who were initially unwilling to participate. In a village where part of the community refused to participate, district health personnel identified the key people who influenced others not to participate and made a special effort to answer their questions and correct their misconceptions. Some of these people went on to become excellent advocates for the study by motivating others to participate.

Physicians played an essential role in the study, especially in the assessment and management of adverse events after treatment. An additional benefit to having physicians on the field team was their ability to examine people with illnesses in participating households and prescribe treatments for common ailments. People with illnesses that required more detailed evaluation or management were referred with written notes to the district hospital. These services increased villagers’ acceptance of our study teams, as early participants reassured their neighbors and encouraged them to participate in the study. The local district health department also supported our work by stationing an ambulance on call at the district hospital to transport participants from the study villages in case they experienced adverse events from the medication that required hospital admission. Fortunately, none occurred. Medical doctors in the government district hospital were also informed about our study and were prepared to manage adverse events. In addition, community support was enhanced through the timely sharing of test results, such as filarial antigen and microfilariae tests, with participants and family members. Field teams visited households in the evening, when most family members were at home. All field research teams included local accredited social health activist workers and primary health center staff. Teams gave biscuits to children who had not eaten recently to improve their ability to tolerate the medications. In addition to door-to-door visits by team members to monitor and manage adverse events, a medical doctor and a local male nurse stayed overnight in each study village on treatment nights. The villagers received phone numbers to call to obtain help for any participant who experienced a significant adverse event after the drug treatment.

### Challenges and responses.

Social unrest in Karnataka state in September 2016 delayed training and initiation of the study for several weeks. In addition, social mobilization was a challenge for the study despite activities mentioned earlier. In addition, our team faced several operational challenges during the study. For example, Internet connection and cell phone coverage were weak in the study area, which affected the electronic reporting of data. Data entry clerks in the field used tablet devices preloaded with electronic data capture software to enter enrollment and follow-up data in real time. CRO personnel synchronized the data every day (from an office in Yadgir town and their headquarters office in Bangaluru), uploaded encrypted data to cloud servers, and worked with VCRC to resolve data queries highlighted by the DOLF data manager in St. Louis, MO.

Some of the study villages were located far from the study headquarters in Yadgir town (on average 40 km), and treatment teams often had to stay in study villages after 11 pm to complete treatment of antigen-positive participants after collecting blood smears. Transport back to their homes was provided for staff who worked in villages at night, and this was welcomed by all.

### Key insights.

The ICMR (both the main office in New Delhi and ICMR-VCRC based in Puducherry) were responsible for overall coordination of the study. The NVBDCP managers at the national and state levels provided important support, despite challenges posed by widespread dengue outbreaks in 2017. Effective communication between the ICMR-VCRC, other stakeholders in India, the WHO South-East Asia Office in India, the Gates Foundation office in India, and DOLF helped coordinate and accelerate the study process. Other important factors included flexibility with rapid adjustments to field procedures based on input from research teams, the electronic data capture system, and timely feedback from the CRO and DOLF in St. Louis were important for clearing data queries. However, the most important key to the success of the study was the dedication of investigators and research staff who worked 7 days per week on rotation to complete the work in a short time.

India’s participation as a study site for the large multicenter IDA safety study accelerated the government’s acceptance of IDA for use in India’s LF elimination program shortly after the regimen was endorsed by the WHO. ^7^ India has been a world leader for implementing MDA with IDA; approximately 50 million treatment doses were distributed in the country between 2018 and 2019. ^4^

## INDONESIA

### Background and preparation for field studies.

Indonesia has the world’s third largest national LF burden, after India and Nigeria. ^4^ Unlike most other endemic countries, the majority of LF in Indonesia is caused by *Brugia malayi* and *B. timori*. This biological difference—together with the fact that Indonesia is the world’s largest archipelago with more than 6,000 inhabited islands spread across more than 3,000 miles—make LF elimination in the country especially challenging. However, DOLF considered other factors to further justify Indonesia’s inclusion in the multicenter IDA safety and efficacy study: the national LF elimination program has made excellent progress in some districts, whereas other districts have struggled; an estimated 38 million people in 118 districts received treatment of filariasis through MDA programs in 2019; the filariasis research team at the Universitas Indonesia has an excellent track record for conducting high-quality field studies.

LF-endemic districts in eastern Indonesia on Sumba and Flores islands were chosen for the study, because their populations had not yet received MDA for filariasis, and because pilot surveys for microfilaremia in adults had shown prevalence in the range of 3–5%. The study area in Sumba was especially remote; it required 2 days to travel there from Jakarta, with several flights and a long overland journey. Because IDA had not been previously used to treat *Brugia* infections, we performed a pilot hospital-based clinical trial to compare the tolerability of IDA versus DA in microfilaremic subjects before proceeding to the larger community MDA study. ^8^ This meant that *B. timori*–infected subjects had to be identified, consented for participation in a clinical trial, and transported by vehicle for 2 hours to the district hospital where they would spend several days away from their families, farms, and fishing grounds.

### Implementation of field studies.

The team took residence in a primary healthcare center, installed satellite Wi-Fi, and secured electricity and potable water for the team. It was especially important for the research team to work together with the Bupati (regent) and district officials and with local health center staff, because this helped them to build trust with the local population. Such trust is essential for obtaining good compliance for night blood surveys, treatment, and posttreatment adverse event assessments. After the pilot study had shown that IDA was just as well tolerated as DA, *dusuns* (hamlets within villages) were randomly assigned to receive MDA with either IDA or DA for the community study. Surveys and treatment were conducted at night, because villagers were often away from their houses during the day and because *B. timori* microfilariae exhibit nocturnal periodicity. Some study villages were accessible only by foot. The study team contacted village residents and local authorities during daylight hours and invited residents to participate in night blood surveys and antifilarial treatment. The research team worked closely with local authorities and health staff to ensure good compliance for night blood surveys, treatment, and posttreatment adverse event assessments.

### Challenges and responses.

Indonesia’s LF elimination program had already provided several rounds of MDA to most endemic areas in the country that had good access and health infrastructure. However, the areas most likely to benefit from IDA are often difficult to access, have limited infrastructure, and require careful social mobilization. Because many villages had no electricity, study teams prepared survey stations during the day. Although the teams sometimes used generators to power lights, they also used headlamps when generator fuel was in short supply. Researchers from Jakarta and DOLF slept in rooms at a primary health center, because there were no hotels or large guest houses in study villages in southwestern Sumba. The team encountered indoor snakes and had to check for scorpions in their shoes during the rainy season. A large tank was installed to hold water for use by the staff and for study procedures. Satellite Wi-Fi was installed to enable data transfer and real-time reporting of adverse events.

### Key insights.

The study in Indonesia enrolled almost 4,000 participants and generated excellent safety and efficacy data. IDA was well tolerated and much more effective for clearing *B. timori* microfilaremia than DA. The fact that a pivotal IDA tolerability study was performed in Indonesia (and the favorable results of the study) gave the Indonesian Ministry of Health confidence to use IDA in districts that had not achieved LF elimination targets after several rounds of MDA with DA; IDA was rolled out in several districts in 2020.

## HAITI

### Background and preparation for field studies.

Haiti is one of only four countries in the Americas with ongoing transmission of LF. ^4^ The Haitian LF elimination program (led by the Ministry of Public Health and Population [MSPP]) first achieved full geographic coverage for MDA with DA in 2012. The MSPP and partners involved in Haiti’s LF elimination program were keen to participate in the multicenter IDA safety study, because IDA has the potential to accelerate LF elimination in the country. As of 2021, 18 of 140 (12.9%) districts in Haiti that were considered endemic for LF based on mapping performed in 2001 had not yet satisfied the WHO criteria for stopping MDA, despite the fact that all had completed at least five rounds of MDA.

The community IDA study in Haiti aimed to enroll 6,000 participants—3,000 in the DA group and 3,000 in the IDA group. The site selected for the study was the Commune of Quartier Morin in the Northern Department of the country. This Commune was known to have persistent LF despite seven prior rounds of MDA with DA. The MSPP led the study, but multiple technical partners assisted with preparation and implementation. The U.S. Centers for Disease Control and Prevention (CDC) (with help from DOLF) provided technical assistance on all aspects of the study, namely protocol development, protocol clearance, training, study implementation, and laboratory analyses. IMA World Health (IMA), a U.S.-based nongovernmental organization and a long-time technical partner of the MSPP’s LF Elimination Program, was selected as the primary implementation partner for the study. The close collaboration between the MSPP and IMA contributed to the success of the study. Study staff were hired by IMA with input from local health authorities in the Northern Department. IMA also provided technical expertise and handled logistics for the study.

The protocol was approved by the National Bioethics Committee of the MSPP. The study team included mostly early career nurses and laboratory technicians with limited research experience. These young and eager professionals were trained on all aspects of the protocol. The training emphasized GCP (especially informed consent, rights to refuse or withdraw from the study, and confidentiality of participants’ information). Training also emphasized the proper assessment and management of adverse events. Staff also received training on how to use the electronic data capture system that was used to manage data across all five IDA safety study sites.

### Implementation of field studies.

New users found the data capture system difficult to use at first. Staff from CDC, IMA, DOLF, and MSPP provided daily supervision of field teams. Daily meetings were scheduled at the end of the day between supervisors and field teams to troubleshoot problems encountered during enrollment and to help resolve challenges faced by teams in the community. Potentially serious and severe adverse events were evaluated by physicians specifically trained for the study who were based at two local hospitals located near the study site. The strong collaboration between field teams and these physicians and hospitals enabled prompt identification and management of the three serious adverse events that were recorded in Haiti. Participants with mild adverse events received care from study nurses in their homes.

### Challenges and responses.

The study faced several significant challenges. The most important of these was reluctance of community members to participate in the study. This was mainly due to MDA fatigue after seven prior rounds. Study procedures helped to counter this reluctance, and study participants expressed genuine appreciation for the professionalism of the study team members who were clearly identified by blue shirts. Some members of the community were impressed with the quality of care provided to participants who experienced adverse events and the time that staff spent answering questions during the consent process. The study team also initially encountered technical challenges such as the lack of a stable, business-level Internet connection that was capable of uploading large amounts of data to the central cloud server for daily synchronization and remote data quality control by the DOLF data manager. Other challenges included multiple interruptions in enrollment related to national elections and a particularly heavy rainy season that resulted in severe flooding of the study area. Despite difficult working conditions, the study teams successfully completed the enrollment and adverse events assessment portions of the study for 6,000 participants in 4 months.

### Key insights.

The Haiti study made a significant contribution to the global IDA safety and efficacy study both in terms of the number of participants enrolled and practical learnings related to MDA with IDA. ^9^ The study helped the MSPP gain experience with IDA that will help them complete Haiti’s LF elimination program, and it strengthened local research capacity, which will serve the MSPP well for future public health activities in the Northern Department.

## PAPUA NEW GUINEA

### Background and preparation for field studies.

Clinical trials in East Sepik Province, Papua New Guinea, had shown that IDA was superior to DA for achieving long-term clearance of *Wuchereria bancrofti* microfilaremia. ^2^^,^ ^3^ A large community study was then required to compare the tolerability and efficacy of IDA and DA when they were used for MDA. Bogia district was selected as the setting for the multicenter IDA safety study in Papua New Guinea, because it was accessible by land from the PNG Institute for Medical Research station located about 4 hours south, near Madang. The coastal road was fairly well-maintained by Papua New Guinea standards, and that enabled access for senior staff from the Institute who were needed to supervise the study. Bogia district had not previously received MDA for LF, and communities and local officials were willing to participate in the study.

### Implementation of field studies.

The coastal villages were scattered along the shore of the Bismarck Sea with palm trees framing white sand beaches. On clear nights, we could see spurts of bright red lava from the active volcano on Manam Island near Bogia. The study team stayed at a guest house in a centrally located village. Because electricity was not available in the village, we used generators for light in the residence and for night surveys across the study area.

### Challenges and responses.

The team faced various challenges related to communications, logistics, human resource management, and community relations. The first major challenge was a cell tower failure in our headquarters village that knocked out Internet access and phone service. We often had to walk to another village some 35 minutes away to get reception. Because reliable communication was a priority for this study, the team contracted for costly satellite-based Internet service. This took time, and unfortunately service was still intermittent. Then an important highway bridge collapsed when an overloaded truck attempted to cross it. This required us to use small boats to get supplies or personnel across the river. A bamboo pontoon foot-bridge was hastily constructed, but it could not be used for heavy supplies, petrol and food that were regularly sent from Madang. The staff also constructed rafts that could be paddled across the river. Fortunately, we were able to transport our vehicles to the other side of the river via a long detour route that led to a shallow part of the river where trucks could cross.

Malaria surged in Bogia district during the study because of a local shortage of antimalarial drugs. This stressed the health center in our study area in ways that compromised our work. Most field staff members experienced one or more bouts of malaria during the study despite our provision of insecticide-treated bed nets and antimalarial prophylaxis. Communal tensions represented another challenge for the study. Many residents of Manam Island resettled in Bogia district because of volcanic eruptions some 10 years prior to our study. Some people in Bogia alleged that the government had not adequately compensated local residents for land taken to accommodate the migrants from Manam. Tensions between long-term residents and migrants sometimes erupted in ways that adversely affected our study. In addition, misinformation about LF and our study was rife in some villages, despite vigorous social mobilization and LF awareness efforts. This misinformation slowed down our work, because staff had to spend additional time repeating awareness messages in some communities where traditional beliefs about the causes of LF led to rumors and distrust.

### Key insights.

These sorts of challenges are standard fare for field research in Papua New Guinea. Fortunately, dedicated staff from the PNG Institute for Medical Research were able to work through these difficulties. They accomplished their mission by enrolling more than 4,500 study participants. The study in Bogia district study showed that IDA was very well tolerated and significantly more effective than DA for clearing microfilaremia. Results from the study provided strong support for the government’s decision to roll out IDA in selected provinces of Papua New Guinea starting in 2019.

## FIJI

### Background.

Starting in the early twentieth century, research conducted in Fiji has contributed to our global understanding of the epidemiology of LF and the role of community-based treatments for LF control. ^10^^,^ ^11^ This history paved the way for Fiji’s participation in the global IDA community safety and efficacy studies. In addition, the study in Fiji offered an opportunity to assess the safety and impact of MDA with IDA versus DA for LF in a setting where scabies and soil-transmitted helminths are co-endemic.

### Preparation for field studies.

We assembled a large research field team from a network of retired health professionals and experienced research staff. Intense microplanning and last-minute flexibility were critically important for the successful completion of this study. The electronic data collection system contributed greatly to data quality and timely data transfer. The system incorporated field limits to prevent incorrect entries and rules that pointed data entry technicians toward correct answers. Timely data transfer led to rapid identification and clearance of data queries. User testing of the electronic data capture system and user training were crucial prior to commencement of the study.

### Implementation of field trials.

Field studies were performed on the islands of Rotuma and Gau that are 640 and 87 km across the ocean from Suva (Fiji’s capital), respectively. Willingness of active local health staff to participate, and their ability to obtain permission for task shifting, were crucial for ensuring community acceptance and participation in the study. The community appreciated the care taken by the research team who explained the purpose of the study, obtained informed consent, and provided close attention and follow-up to assess and manage adverse events. High population treatment coverage was facilitated by mop-up sessions for those who were missed during the initial enrollment attempt. Use of satellite Wi-Fi in these remote settings enabled daily uploads of data and synchronization of data collected on different days.

### Challenges and responses.

Slow ethics committee approval of the research protocol delayed study initiation by several months. Additional training and mentorship to strengthen the capacity of ethics committee members could help prepare the committee to review future protocols with similar complexity. Travel plans were frequently affected by tropical storms and weak transportation infrastructure in these remote island settings.

This study used a modified version of the multicenter IDA study protocol to allow for the collection of data on the epidemiology, prevalence, and response to treatment of scabies, and soil-transmitted helminths in addition to LF. This additional complexity had only a minor impact on the cost of the project, and the prospect of treating three infections with a single treatment increased community enthusiasm and participation in the study.

### Key insights.

The Fiji IDA tolerability study was successful despite significant challenges. More than 3,400 participants were enrolled, treated, and followed-up for assessment of adverse events. ^12^ The Fijian National LF program was able to review the WHO recommendation ^13^ to adopt IDA with the knowledge and confidence that the local safety profile of IDA was comparable to the well-tolerated DA, which has been the standard regimen for LF control in Fiji for over 10 years.

## CONCLUSION

This article includes interesting experiences and important insights from studies that were conducted in diverse and difficult settings. The challenges described in these stories can be thought of as variations on several themes (difficult logistics, communications and Internet connectivity, regulatory hurdles, community relations, and staffing). Fortunately, resourceful research teams found ways to overcome these challenges. At the end of the day, these studies provided high-quality data that helped the transition of IDA from clinical trial to policy. Lessons from these reports have broad validity, and we hope that they will help others who wish to evaluate new interventions for neglected tropical diseases.
